# Isoproterenol enhances force production in mouse glycolytic and oxidative muscle via separate mechanisms

**DOI:** 10.1007/s00424-019-02304-0

**Published:** 2019-08-21

**Authors:** Sarah J. Blackwood, Abram Katz

**Affiliations:** Åstrand Laboratory of Work Physiology, Swedish School of Sport and Health Sciences, GIH, Box 5626, S-114 86 Stockholm, Sweden

**Keywords:** Isoproterenol, Muscle, Force, Fatigue, Metabolites, Phosphorylase

## Abstract

Fight or flight is a biologic phenomenon that involves activation of β-adrenoceptors in skeletal muscle. However, how force generation is enhanced through adrenergic activation in different muscle types is not fully understood. We studied the effects of isoproterenol (ISO, β-receptor agonist) on force generation and energy metabolism in isolated mouse soleus (SOL, oxidative) and extensor digitorum longus (EDL, glycolytic) muscles. Muscles were stimulated with isometric tetanic contractions and analyzed for metabolites and phosphorylase activity. Under conditions of maximal force production, ISO enhanced force generation markedly more in SOL (22%) than in EDL (8%). Similarly, during a prolonged tetanic contraction (30 s for SOL and 10 s for EDL), ISO-enhanced the force × time integral more in SOL (25%) than in EDL (3%). ISO induced marked activation of phosphorylase in both muscles in the basal state, which was associated with glycogenolysis (less in SOL than in EDL), and in EDL only, a significant decrease (16%) in inorganic phosphate (P_i_). ATP turnover during sustained contractions (1 s EDL, 5 s SOL) was not affected by ISO in EDL, but essentially doubled in SOL. Under conditions of maximal stimulation, ISO has a minor effect on force generation in EDL that is associated with a decrease in P_i_, whereas ISO has a marked effect on force generation in SOL that is associated with an increase in ATP turnover. Thus, phosphorylase functions as a phosphate trap in ISO-mediated force enhancement in EDL and as a catalyzer of ATP supply in SOL.

## Introduction

Fight or flight is an established biologic response to a potentially harmful event or perceived threat to survival [16]. It entails a sympathetic discharge consisting of adrenaline (AD) release from the adrenal medulla (as well as noradrenaline from sympathetic nerves) that affects different organs and pathways in the body. With respect to skeletal muscle, AD binds to β-adrenoceptors and enhances force production in skeletal muscle. Inhibition of β-adrenoceptors (e.g., with propranolol) abolishes the effects of AD and sympathomimetics on muscle function [9, 19, 29, 31, 60]. Indeed, sympathomimetic agents increase force generation specifically via β_2_ receptors [9]. The mechanism by which sympathomimetic agents enhance muscle performance has not been fully elucidated [9, 11, 39], but evidence indicates that force augmentation during tetanic contractions occurs via increases in myoplasmic-free Ca^2+^ concentration ([Ca^2+^]_i_) [3, 11, 15, 30]. The latter depends on cyclic AMP-dependent protein kinase (PKA)–mediated phosphorylation of ryanodine receptor 1 (serine residue S2844), which results in Ca^2+^ release from the sarcoplasmic reticulum (SR) [3].

Often, fight or flight lasts between several and ~ 30 s. Under such conditions, it appears that β_2_-adrenergic activation induces a delay in skeletal muscle fatigue [14, 35, 39]. However, the mechanisms responsible for the enhancement of muscle force generation under such conditions are not fully understood, nor is it known whether the mechanisms are the same in different muscle fiber types. For example, adrenaline activates phosphorylase and stimulates glycogenolysis (albeit to a low extent compared with exercise) to a greater extent in glycolytic (primarily type II) vs. oxidative (primarily type I) muscle fibers at rest [17, 28, 57]. This would be expected to result in a greater accumulation of hexose monophosphates and a larger decrease in inorganic phosphate (P_i_) in glycolytic fibers. Since P_i_ inhibits cross-bridge function [2, 21, 50], one would expect this mechanism to be more prominent in enhancing muscle performance in glycolytic fibers. Thus, the purpose of the present study was to assess the effects of isoproterenol (ISO, β-adrenoceptor agonist) on force production, force × time integral, and metabolism in isolated mouse soleus (SOL, oxidative) and extensor digitorum longus (EDL, glycolytic) muscles under stimulation conditions that result in maximal force generation. Isolated muscles were studied to avoid confounding effects of other factors that could affect muscle function (e.g., changes in blood flow). The results indicate that under conditions of maximal stimulation, ISO enhances force production and force × time integral in both SOL and EDL, but more in SOL. The increased force in SOL is associated with an increased ATP turnover, whereas the increased force in EDL is associated with a decrease in the muscle content of P_i_.

## Materials and methods

### Animals and materials

Adult male mice (C57Bl/6JOlaHSd, *n* = 50) aged 10–14 weeks (~ 25 g) were housed at room temperature (22 °C) on a 12:12-h light-dark cycle. Food and water were provided ad libitum. Animals were killed by cervical dislocation, and EDL and SOL muscles were isolated. Previous studies have established that EDL muscles of C57Bl6 mice express only fast myosin isoforms (~ 80% IIb, ~ 20% IIx), while SOL muscles express slow (~ 40% I) and fast (~ 55% IIa; ~ 5% IIx) myosin isoforms [40, 46]. These myosin distributions indicate that EDL muscle fibers are primarily glycolytic, whereas the SOL muscle fibers are primarily oxidative [52]. The observation that the maximal mitochondrial respiration rate of saponin-skinned mouse soleus fibers is approximately twofold that of EDL fibers supports this conclusion [44]. All experiments were conducted in accordance with the Guide for the Care and Use of Laboratory Animals and were approved by the Ariel University animal ethics committee. All reagents, (−)-isoproterenol hydrochloride (ISO), and enzymes were from Sigma or Roche, except for D-[U-^14^C]-glucose-1-P and uridine diphosphate (UDP)-[U-^14^C]-glucose, which were purchased from Perkin-Elmer.

### Mounting, solution, and stimulation

For stimulation experiments, silk sutures were tied to tendons and muscles mounted in a stimulation chamber (World Precision Instruments, FL, USA). One tendon was attached to a fixed hook and the other to a force transducer with an adjustable holder (allowing alteration of muscle length). Muscles were bathed in a Tyrode solution containing (in mM): 121 NaCl, 5 KCl, 1.8 CaCl_2_, 0.5 NaH_2_PO_4_, 0.4 MgCl_2_, 24 NaHCO_3_, 0.1 EDTA, and 5.5 glucose and constantly gassed with 5% CO_2_:95% O_2_, giving a final pH of 7.4. Chamber temperature was constantly maintained at 30 °C by a water jacketed circulation bath [61]. Muscles were stimulated electrically with platinum electrodes lying parallel to muscles using current pulses of 0.5 ms duration (150% of the current required to elicit maximal force). After mounting, muscles were allowed to recover from dissection for 10 min, and optimal length for generation of maximal isometric tetanic force was set with fused tetanic isometric contractions (100 ms trains at 70 Hz).

### Protocol 1 — force frequency curves and continuous contraction

**Fig. 1:**
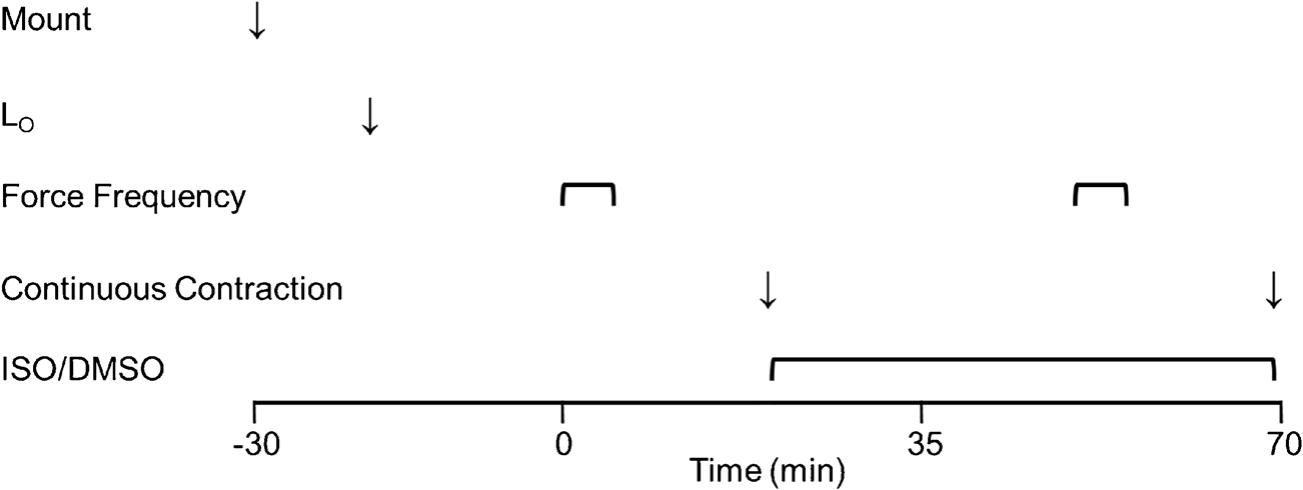
Scheme for Protocol 1. After setting optimal length (L_O_), EDL muscles equilibrated for 20 min, followed by baseline force frequency and a prolonged (continuous) contraction. Thereafter, ISO (or DMSO) was administered for 30 min before force frequency and continuous contraction was repeated. The scheme looks the same for soleus muscles with the exception that after setting L_o_, equilibration was for 25 min

After an equilibration period (25 min SOL and 20 min EDL), a force frequency curve was performed followed by one sustained contraction (see below). Paired SOL and EDL muscles were stimulated at 1-min intervals using 600 ms (SOL, 1–100 Hz) or 300 ms (EDL, 1–150 Hz) trains. Muscles then rested for 15 min and were subsequently stimulated to perform one sustained contraction (SOL, 100 Hz, 30 s; EDL 120 Hz, 10 s). Thereafter, muscles recovered for 30 min either in the presence of 10 μM ISO (diluted in dimethyl sulfoxide, DMSO) or in an equivalent volume of DMSO as a control (CON; final concentration = 0.04%). Force frequency curves and sustained contractions were then repeated (see above) in the presence or absence of ISO. The ISO concentration and incubation duration employed should yield maximal effects [24, 38, 56]. The protocol is presented in schematic form in Fig. 1.

### Protocol 2 — EDL force frequency curve

**Fig. 2:**
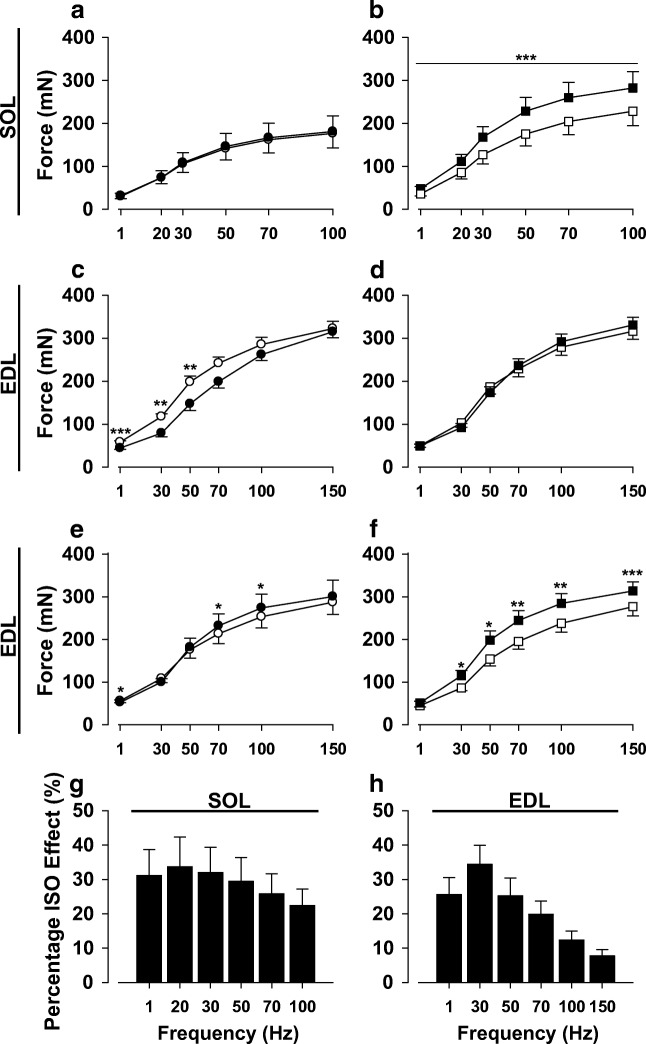
Isoproterenol enhances force generation of soleus and extensor digitorum longus muscles. Force frequency curves were performed at baseline (○, □) and 30 min post-treatment for control (●) and isoproterenol (■) groups in soleus (SOL) (**a**, **b**) and extensor digitorum longus (EDL) (**c**−**f**), respectively. Forces are substantially higher than baseline in the presence of ISO in SOL (**b**). After baseline, a prolonged contraction was performed in each muscle (see Fig. 3), which caused a rundown effect in the EDL muscle with CON values significantly decreasing from baseline (**c**). Consequently, there were only minor increases in force in the presence of ISO (**d**). Force frequency curves were therefore performed in another series of EDL muscles without continuous contraction after baseline, CON (**e**) and ISO (**f**). In the latter case, forces are clearly higher than baseline in the presence of ISO (**f**). The corresponding average percentage effect of ISO on force is shown for SOL (**g**) and EDL (**h**) muscles. **h** is derived from results from **d** and **f**, with adjustments for rundown in **d**. Data are expressed as mean ± SE for 5 (**e**−**f**), 8 (**a**−**d** and **g**), or 13 (**h**) muscles. *p < 0.05; **p < 0.01; ***p < 0.001 vs. corresponding treatment by paired *t* test

Protocol 1 produced a rundown effect in the EDL muscles, with CON treatment values lower at a number of contraction frequencies compared to baseline (Fig. 2c), as has been previously reported at 24–27 °C in fast-twitch muscle preparations [13, 56]. Force frequency curves were therefore repeated on paired EDL muscles (before and after addition of ISO or DMSO) without a sustained contraction, as described above.

### Protocol 3 — stimulation for biochemical analysis

Paired EDL and SOL were allowed to recover for 20 (EDL) or 25 (SOL) min after setting optimal length. Thereafter, and following a single control tetanus (600 ms train, 100 Hz for SOL; 300 ms train 120 Hz for EDL), ISO or DMSO (CON) were added to the medium for 30 min (see above). In initial experiments, NaCN (final concentration = 3 mM) was added to the medium after 25 min of incubation in ISO or DMSO (to inhibit cytochrome oxidase to create an anoxic environment, thereby enabling estimates of ATP turnover). In subsequent experiments, NaCN was omitted (see “Results”). Muscles were then stimulated to perform one sustained contraction (as in protocol 1) for 1 (EDL) or 5 s (SOL) and the muscles were subsequently rapidly frozen in liquid N_2_. Non-stimulated controls were frozen at the same times of incubation. The intent of these experiments was to correlate metabolic changes with the enhancement of force by ISO.

### Analytical

Force signals were recorded online with LabScribe2 software (iWorx, NH, USA) and stored on a personal computer for subsequent analysis. Peak tension was measured during tetani. Time of force development and relaxation time were estimated as time to 50% of peak tension (½ TTP) and time to 50% relaxation (½ RT) [43]. For analytical biochemistry, muscles were freeze-dried and freed from non-muscle constituents. Muscle tissue was powdered, thoroughly mixed, and processed further (for glycogen) or divided into two aliquots (for enzymes and metabolites). One aliquot was extracted in ice-cold 0.5 M perchloric acid, centrifuged, neutralized with 2.2 KHCO_3_, and centrifuged again. The final supernatant was analyzed for metabolites using enzymatic techniques (changes in NAD[P]H) adapted for fluorometry [45]. To correct for variability in solid non-muscle constituents, metabolite values were adjusted for total creatine (TCr). ISO did not significantly affect TCr under any condition studied. TCr averaged 73.8 ± 1.6 μmol/g dry weight in SOL and 128.8 ± 2.4 μmol/g dry weight in EDL. For analysis of glycogen, muscle was digested in hot (80 °C) 1 N NaOH and hydrolyzed with amyloglucosidase to free glucose [42], which was converted to NADPH and analyzed fluorometrically [45]. Aliquots of extract were analyzed for protein (BioRad method) and glycogen values were adjusted for protein. ISO did not significantly affect protein under any condition studied and protein averaged 357 ± 5 μg/mg dry weight in EDL and 326 ± 7 μg/mg dry weight in SOL.

Anaerobic ATP turnover was calculated as follows: − 2∆ATP − ∆PCr + (1.5∆lactate) [42], where ∆ is the mean contraction value minus the mean basal value (Table 3). In using this formula, it is assumed that during short-term intense contractions all lactate production is from glycogen and there is no loss of lactate into the medium [42]. In experiments where NaCN was omitted, aerobic ATP production during contraction was estimated using published O_2_ consumption values of isolated mouse SOL (4 μL/s/g wet weight) and EDL (13.5 μL/s/g wet weight) [5] (adjusting for temperature differences by assuming a Q_10_ of 2) and myoglobin concentration of oxidative (3.15 mg/g wet weight) and glycolytic (0.5 mg/g wet weight) rodent skeletal muscle [33], assuming a myoglobin molecular mass of 16.95 kDa and a myoglobin:O_2_ ratio of 1:1, a P/O_2_ of 6 and 4.3 g wet weight/g dry weight.

The second aliquot was homogenized (200 μL/mg dry weight) with a ground glass homogenizer in an ice-cold buffer of (in mM): 10 EDTA, 50 KF, and 30% glycerol (v/v), pH 7.0, and centrifuged. The supernatant was diluted and used for analysis of glycogen phosphorylase (Phos) and glycogen synthase (GS) activity following the incorporation of D-[U-^14^C]glucose-1-P into glycogen and UDP-[U-^14^C]glucose into glycogen, respectively, as previously described. Total and fractional activities (± 3 mM AMP for Phos; 0.17/7.2 mM G6P for GS) were measured as described elsewhere [42], with the exception that glucose 1-P concentration was 67 mM in the absence and presence of AMP. Total activities were adjusted for TCr as above.

### Statistics

Values are given as mean ± SE. Statistical significance was set at *P* < 0.05 and was determined with paired or unpaired *t* tests. Results from muscles that produced low forces were excluded.

## Results

### Effects of isoproterenol on contractile properties of SOL and EDL muscles

**Fig. 3:**
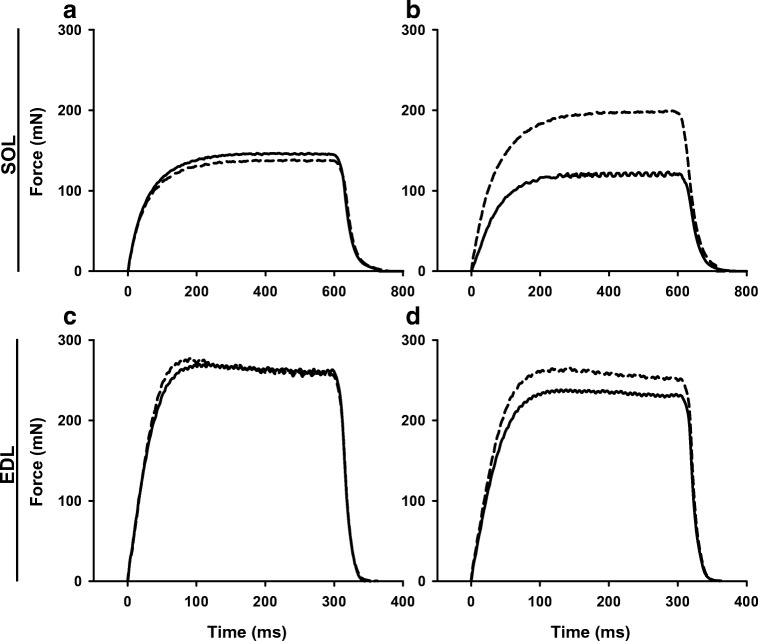
Representative force recordings for soleus and extensor digitorum longus muscles during maximal stimulation frequencies. Forces are shown for a given muscle before (solid line) and after (dashed line) treatment. Soleus muscles were stimulated at 100 Hz before and after exposure to diluent (**a**) or ISO (**b**). Extensor digitorum longus (EDL) muscles were stimulated at 150 Hz before and after exposure to diluent (**c**) or ISO (**d**). Note that at these frequencies ISO enhanced force markedly more in soleus than in EDL

Exposure to ISO-enhanced force generation in SOL muscles at all stimulation frequencies, while similar force values were obtained before and after addition of DMSO (Fig. 2a, b). In contrast, ISO resulted in increased forces only at high stimulation frequencies in EDL (Fig. 2d); however, a rundown effect was noted in the control group (decrease in force during second force frequency run), especially at the lower stimulation frequencies (Fig. 2c). Therefore, an additional series of experiments was performed where prolonged continuous tetani were omitted. Under these conditions, rundown was abolished and an ISO effect on force was seen at all stimulation frequencies (Fig. 2e and f). Estimates of ISO-mediated force enhancement showed a robust effect in SOL at all stimulation frequencies (Fig. 1g). In the EDL, force enhancement was substantial at lower frequencies but less so at the higher frequencies (Fig. 1h). Thus, the peak ISO effect occurred at low frequencies in both SOL (33.7 ± 8.7% at 20 Hz, Fig. 1g) and EDL (34.4 ± 5.6% at 30 Hz, Fig. 1H), whereas for maximal tetanic contractions, the ISO effect was better maintained in the SOL (22.4 ± 4.8% at 100 Hz) than in the EDL (7.7 ± 1.8% at 150 Hz) (*P* < 0.01 for SOL vs. EDL). The latter is clearly seen in representative force recordings for both EDL and soleus muscles (Fig. 3). The ½ time to peak tension (TTP) was slightly faster after ISO treatment in SOL at the higher frequencies, but a similar observation was observed in the control condition at 100 Hz (treatment with DMSO vs. baseline). Therefore, these changes are not considered to be of physiologic significance (Table 1).

**Table 1:** Effects of isoproterenol on contractile properties in soleus and extensor digitorum longus muscles

Stimulation frequency		Time to 50% of peak tension (ms)				Time to 50% of decrease of tension (ms)			
		Control		Isoproterenol		Control		Isoproterenol	
		Baseline	Treatment	Baseline	Treatment	Baseline	Treatment	Baseline	Treatment
SOL	1 Hz	6.6 ± 0.2	6.6 ± 0.4	5.9 ± 0.2	6.4 ± 0.2	37.9 ± 3.6	33.0 ± 1.7	31.0 ± 1.5	29.6 ± 1.2
	20 Hz	54.0 ± 4.9	57.3 ± 0.6	47.5 ± 7.4	47.9 ± 7.0	44.8 ± 1.8	45.8 ± 2.7	47.8 ± 3.4	43.1 ± 2.7*
	30 Hz	56.9 ± 4.4	53.5 ± 4.5	60.5 ± 4.9	57.0 ± 4.8	42.6 ± 1.6	43.0 ± 1.3	43.8 ± 4.0	42.1 ± 1.4
	50 Hz	55.0 ± 2.1	53.0 ± 2.5	52.9 ± 2.6	50.6 ± 2.0*	41.3 ± 1.2	44.8 ± 2.2	42.3 ± 2.0	39.6 ± 1.1
	70 Hz	51.1 ± 2.0	49.8 ± 1.9	49.5 ± 1.8	46.9 ± 1.7*	41.5 ± 1.3	42.9 ± 1.7	41.1 ± 2.5	40.9 ± 1.3
	100 Hz	46.9 ± 1.8	44.9 ± 1.4*	44.9 ± 1.7	42.8 ± 1.6**	44.5 ± 1.6	46.6 ± 2.2	46.0 ± 2.1	45.0 ± 1.7
EDL	1 Hz	5.0 ± 0.3	4.6 ± 0.2	4.2 ± 0.2	4.4 ± 0.2	24.6 ± 1.3	20.0 ± 2.7	24.8 ± 1.2	21.6 ± 2.1
	30 Hz	16.0 ± 5.3	20.4 ± 6.4	10.8 ± 0.8	22.4 ± 6.2	14.4 ± 0.7	12.4 ± 1.4	14.2 ± 1.5	12.8 ± 0.7
	50 Hz	25.8 ± 0.5	28.0 ± 1.7	29.6 ± 3.1	31.6 ± 3.0	15.4 ± 1.2	15.4 ± 1.9	14.4 ± 1.4	18.0 ± 1.7
	70 Hz	25.8 ± 2.0	28.0 ± 2.5	31.0 ± 0.7	32.0 ± 0.9	16.2 ± 0.6	18.0 ± 1.0	15.8 ± 0.7	18.6 ± 1.4*
	100 Hz	25.0 ± 1.1	26.0 ± 1.8	27.8 ± 1.7	28.2 ± 1.2	17.8 ± 0.7	19.6 ± 0.7**	16.6 ± 0.7	19.0 ± 0.9
	150 Hz	22.6 ± 1.0	23.6 ± 1.2	24.8 ± 1.4	24.8 ± 0.8	19.6 ± 0.9	20.8 ± 1.0*	20.2 ± 1.0	22.4 ± 3.0

Results are obtained from force frequency curves where solei (SOL) were stimulated with a single 600-ms train and extensor digitorum longus (EDL) muscles were stimulated with a single 300-ms train. Data are expressed as mean ± SE for five (EDL, no rundown muscles) or eight (SOL) muscles

*p < 0.05

**p < 0.01 vs. corresponding baseline value by paired *t* test

### Effects of isoproterenol on force × time integral in SOL and EDL muscles

**Fig. 4:**
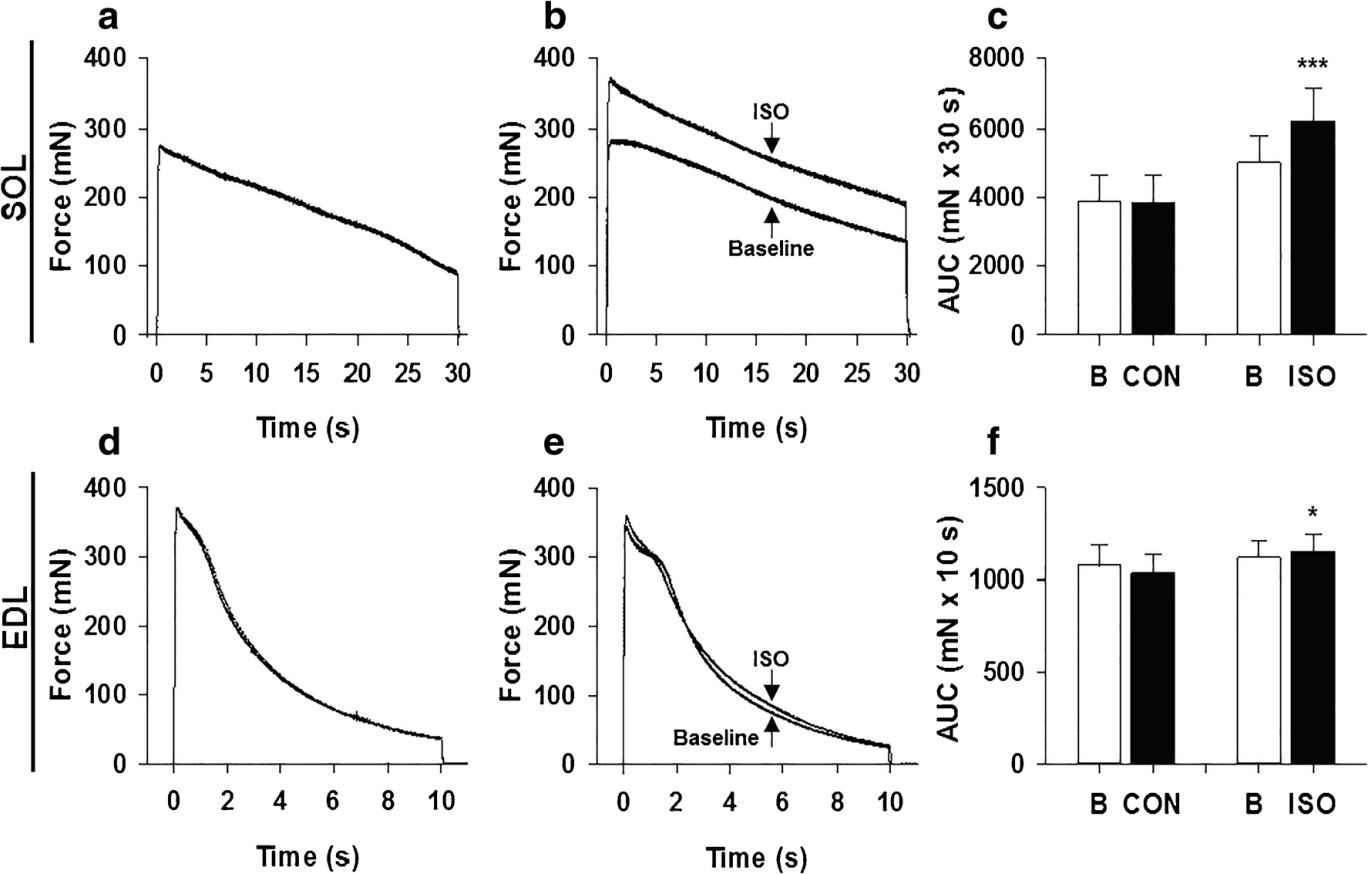
Isoproterenol enhances work output during prolonged contractions of soleus and extensor digitorum longus muscles. Prolonged tetanic contractions were performed for 30 s at a frequency of 100 Hz in SOL and 10 s at a frequency of 120 Hz in EDL. Representative traces are shown for baseline and 30 min post-treatment in CON and ISO groups as indicated for SOL (**a, b**) and EDL (**d, e**), respectively. Note that in **a** and **d**, baseline and CON curves are virtually identical, whereas in panel **b** the ISO curve is markedly higher throughout in comparison to baseline and in **e** the ISO curve is slightly higher initially vs. baseline. The effect of ISO on force × time integral was quantified by determining the area under the curve (AUC) of the continuous contractions in SOL (**c**) and EDL (**f**) muscles at baseline (B, unfilled bar) and 30 min post-treatment (CON and ISO, filled bars). Data are expressed as mean ± SE for eight muscles. *p < 0.05; ***p < 0.001 vs. corresponding baseline values by paired *t* test

Representative traces in Fig. 4 show similar force production during continuous stimulations 30 min apart in CON groups for both SOL (4A) and EDL (4D). In SOL, ISO markedly enhanced force output and this was maintained throughout the 30 s tetanus (Fig. 4b). In EDL, ISO had a minor effect on force generation that was observed primarily at the onset of contraction (Fig. 4e). The force × time integral during contraction was markedly enhanced by ISO in SOL (25.4 ± 4.9%; Fig. 4c), but only slightly in EDL (3.2 ± 1.3%; Fig. 4f) (*P* < 0.01 for SOL vs. EDL).

### ISO and metabolism SOL and EDL muscles

**Fig. 5:**
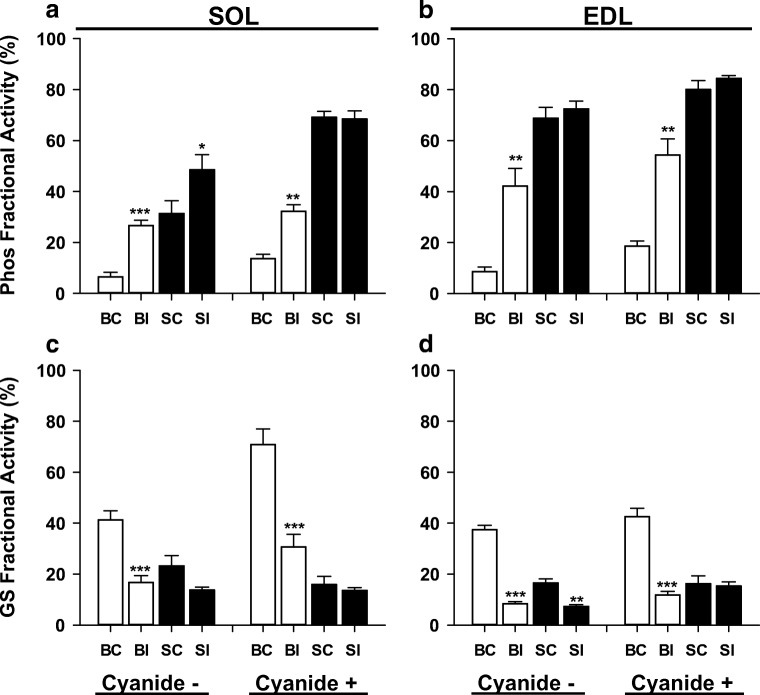
Isoproterenol activates phosphorylase and inactivates glycogen synthase in soleus and extensor digitorum longus muscles. Phosphorylase (Phos) and glycogen synthase (GS) fractional activities were determined in soleus (SOL, **a** and **c**) and extensor digitorum longus muscles (EDL, **b** and **d**). Treatments consisted of basal control (BC), basal isoproterenol (BI), stimulated control (SC), and stimulated isoproterenol (SI) in the absence (−) or presence (+) of 3 mM sodium cyanide. SOL muscles were stimulated for 5 s at 100 Hz and EDL muscles for 1 s at 120 Hz. Data are expressed as mean ± SE for 5–7 muscles (numbers vary as sufficient extract was not always available). *p < 0.05; **p < 0.01; ***p < 0.001 vs. corresponding baseline values by paired *t* test

To assess whether the enhancement of force generation during continuous contractions could be attributed to cellular metabolic changes, muscles were frozen at 1 s (EDL) or 5 s (SOL) of continuous contractions (time points where force was elevated — Fig. 4). Initial experiments were performed in the presence of sodium cyanide (NaCN) to allow for assessment of ATP turnover [42, 61]. Following 30 min exposure to ISO, phosphorylase fractional activity increased to ~ 30% in SOL and to ~ 55% in EDL muscles at rest in the presence of NaCN (Fig. 5a, b). Following a single sustained contraction under control conditions in the presence of NaCN, fractional activity increased to almost 70% in SOL and 80% in EDL, and ISO had no additional effect. As expected, ISO resulted in marked decreases in glycogen synthase (GS) fractional activity in SOL and EDL at rest in the absence or presence of NaCN (Fig. 5c, d). Contraction resulted in marked decreases of GS fractional activity as well, with no further effect of ISO in either muscle.

Basal state measurements of metabolites after 5 min in NaCN showed a marked depletion of phosphocreatine (PCr, ~ 30% in SOL and 60% in EDL) and accumulation of lactate (~ 2-fold in SOL and 13-fold in EDL) (data not shown). These changes in metabolite values precluded accurate measurements of energy turnover. Similar results were previously reported in isolated rat EDL muscle following 10 min incubation in 2 mM NaCN at 30 °C [54]. Therefore, experiments were repeated in the absence of NaCN. With respect to enzyme activities, the results were similar to those observed in NaCN, with some differences. First, basal phosphorylase fractional activity was ~ 50% lower in SOL and EDL in the absence of NaCN (Fig. 4). This is likely explained by a small increase in [Ca^2+^]_i_ as a result of NaCN exposure, which should result in activation of phosphorylase kinase and thereby phosphorylation of phosphorylase **b** [41]. Second, the stimulation-mediated activation of phosphorylase in SOL was only about half that seen in the presence of NaCN (increase to 31% vs. 69% in NaCN), which corresponded with an additive effect of ISO and stimulation on fractional activity. Last, GS fractional activity was markedly lower in SOL in the basal state in the absence of NaCN. Total activities of phosphorylase and GS were not affected by ISO under any condition (Table 2).

**Table 2:** Total phosphorylase and glycogen synthase activities in soleus and extensor digitorum longus muscles in the absence or presence of sodium cyanide

		Cyanide ˗				Cyanide +			
		Basal		Stimulated		Basal		Stimulated	
		Control	Isoproterenol	Control	Isoproterenol	Control	Isoproterenol	Control	Isoproterenol
Phos	*SOL*	69.1 ± 4.1	77.6 ± 5.9	77.9 ± 6.5	62.2 ± 6.8	94.8 ± 7.2	77.3 ± 9.8	89.8 ± 10.6	96.7 ± 8.0
	*EDL*	224.2 ± 19.0	227.2 ± 20.5	250.7 ± 8.6	293.5 ± 25.7	279.0 ± 7.8	265.0 ± 11.0	309.0 ± 7.0	312.0 ± 33.2
GS	*SOL*	3.3 ± 0.4	3.0 ± 0.1	3.5 ± 0.5	2.4 ± 0.6	2.6 ± 0.3	2.9 ± 0.5	3.1 ± 0.2	2.8 ± 0.4
	*EDL*	2.6 ± 0.3	1.9 ± 0.3	1.9 ± 0.3	1.6 ± 0.4	1.9 ± 0.2	1.9 ± 0.2	1.8 ± 0.2	1.4 ± 0.2

Experimental conditions are as in the legend of Fig. 3. Data expressed as mean ± SE and are given in μmol/min/g dry weight for 5–7 muscles (numbers vary as sufficient extract was not always available). Paired *t* test used to determine significance

In the basal state, exposure to ISO for 30 min resulted in a decrease of glycogen of almost 10 μmol glucosyl units/g dry weight in SOL muscle, but this decrease was not statistically significant (Table 3). In the basal condition, ISO resulted in a ~ 5-fold increase in glucose 6-P, but no significant change in lactate. Accumulation of glucose 6-P and lactate accounted for ~ 50% of glycogen breakdown induced by ISO, indicating that approximately half of the glycogenolytic breakdown products were either released (as lactate) or oxidized (as pyruvate) in mitochondria. It should be noted that in the presence of β-adrenergic stimulation, glucose transport is either unaffected or decreased in skeletal muscle [8, 18, 60], indicating that the increase in glycogenolytic intermediates in the presence of ISO is derived from glycogen breakdown and not glucose transport. Other metabolites were not significantly affected by ISO at rest in SOL. However, following contraction, lSO resulted in significantly greater accumulation of lactate and larger depletion of PCr. In the EDL muscle under basal conditions, ISO resulted in a breakdown of ~ 20 μmol glucosyl units/g dry weight, a > 15-fold increase in glucose 6-P, and a significant increase in lactate (accumulation of glycogenolytic intermediates accounted for < 30% of glycogen breakdown), demonstrating a greater activation of glycogenolysis compared to SOL. Additionally, in EDL in the basal state, ISO resulted in a statistically significant decrease in P_i_ (16%). Following contraction, ISO did not significantly affect any of the measured metabolites in EDL. In the stimulated state, glycogen values in the presence of ISO did not differ significantly from control in either muscle. It should be noted, however, that under the present conditions (1 and 5 s contractions for EDL and SOL, respectively), accurate measurements of glycogenolysis are better reflected by accumulation of glycogenolytic intermediates (primarily glucose 6-P and lactate), as discussed elsewhere [42].

**Table 3:** Metabolite contents in soleus and extensor digitorum longus muscles

	*SOL*				*EDL*			
	Basal		Stimulated		Basal		Stimulated	
	CON	ISO	CON	ISO	CON	ISO	CON	ISO
*Glycogen*	70.4 ± 4.8	61.6 ± 5.8	57.6 ± 6.7	56.1 ± 7.9	52.3 ± 4.8	32.5 ± 1.8**	29.3 ± 3.7	25.8 ± 2.4
*G6P*	0.71 ± 0.09	3.94 ± 0.17***	3.75 ± 0.50	4.31 ± 0.36	0.22 ± 0.04	3.79 ± 0.42***	5.93 ± 0.92	6.36 ± 0.62
*Lactate*	6.3 ± 1.0	9.1 ± 1.9	12.5 ± 1.0	17.8 ± 2.0**	1.7 ± 0.3	5.1 ± 0.4**	28.5 ± 5.4	32.7 ± 4.8
*Malate*	0.97 ± 0.12	1.00 ± 0.07	1.66 ± 0.12	1.91 ± 0.17	0.18 ± 0.03	0.26 ± 0.04	0.73 ± 0.10	0.94 ± 0.11
*ATP*	17.4 ± 0.6	17.7 ± 1.6	20.1 ± 1.0	17.1 ± 0.7	28.9 ± 0.7	27.8 ± 0.5	25.9 ± 1.4	27.9 ± 2.4
*Pi*	26.1 ± 2.2	22.5 ± 1.1	34.9 ± 2.2	36.2 ± 2.1	16.9 ± 0.8	14.3 ± 0.6*	47.8 ± 6.3	54.0 ± 8.2
*PCr*	40.9 ± 1.9	39.4 ± 1.8	33.2 ± 3.1	24.6 ± 2.6**	89.2 ± 1.3	87.6 ± 1.5	44.0 ± 6.8	44.2 ± 4.2
*Cr*	28.6 ± 1.9	30.0 ± 1.8	36.1 ± 3.1	44.7 ± 2.6**	20.3 ± 1.3	21.8 ± 1.5	93.1 ± 6.8	85.3 ± 8.4

Data are expressed as mean ± SE. Values are given in μmol/g dry weight (for glycogen μmol glucosyl units/g dry weight), six muscles for metabolites and seven (SOL) and six (EDL) glycogen

*p < 0.05

**p < 0.01

***p < 0.001 vs. CON by paired *t* test

Estimates of non-aerobic ATP turnover during contraction in SOL averaged 11.5 and 29.2 μmol/g dry weight in the absence and presence of ISO, respectively, and total ATP turnover (including utilization of oxygen) averaged 22.2 and 39.3 μmol/g dry weight, respectively. Estimates of non-aerobic ATP turnover during contraction in EDL averaged 91.3 and 84.6 μmol/g dry weight in the absence and presence of ISO, respectively, and total ATP turnover (including utilization of oxygen) averaged 97.3 and 90.6 μmol/g dry weight, respectively. It was assumed that ISO did not affect oxygen consumption under any condition, and this was supported by the finding that malate (a marker of oxidative metabolism [36, 61]) was not affected by ISO under any condition (Table 3). The minor relative contribution of oxygen to total ATP turnover in EDL and significant contribution in SOL is consistent with previous findings under comparable conditions [22]. Thus, ISO markedly increased ATP turnover in SOL during contraction, but was without noteworthy effect in EDL.

## Discussion

The major findings of the present study are that ISO: (1) increases maximal force production and force × time integral more in SOL than in EDL; (2) activates glycogenolysis in the basal state to a greater extent in EDL, but during contraction glycogenolysis is enhanced only in SOL; and (3) decreases P_i_ in the basal state in EDL and increases ATP turnover during contraction in SOL.

In accordance with previous studies [3, 12, 15, 30], we observed that activation of β-adrenoceptors enhances force markedly in SOL and EDL during low stimulation frequencies. Further, we found that the β-adrenergic mediated enhancement of force during high stimulation frequency continues to be robust in SOL but decreases in EDL, in concordance with earlier findings [12, 13, 15]. Additionally, ISO markedly increased the force × time integral throughout a prolonged continuous contraction (30 s) at a high stimulation frequency in SOL (25%), as shown earlier [14]. Finally, ISO also increased the force × time integral throughout a prolonged contraction in EDL (10 s), but the increase was small (< 5%) and appeared to be limited to the first s of stimulation.

It has been shown that β-adrenoceptor agonists increase force generation by enhancing SR Ca^2+^ release (see Introduction) with no noteworthy effect on sensitivity of cross-bridges to Ca^2+^ [11, 15, 30]. The implication from this finding is that β-adrenoceptor activation should not alter cross-bridge function via cAMP-dependent protein kinase (PKA) mediated phosphorylation of proteins that affect force generation [11], nor metabolic alterations such as changes in P_i_ [12]. With respect to the latter, it is well established that conditions resulting in increased myoplasmic P_i_ concentration inhibit force generation, whereas conditions that result in decreases in P_i_ concentration enhance force generation [1, 2]. Noteworthy is that the inhibition of cross-bridge force generation by P_i_ decreases substantially at near physiologic temperature [23, 55]. At 30 °C, P_i_ has little effect on force at very high [Ca^2+^]_i_ (pCa = 5.8) [1]. However, at lower [Ca^2+^]_i_ (pCa = 6.0), the inhibition of force by P_i_ becomes marked, even at 30 °C [1]. Indeed measurements of [Ca^2+^]_i_ in intact mouse muscle fibers under maximal stimulation conditions (with and without activation of β-adrenoceptors) demonstrate values that are close to a pCa of 6.0 [14, 53], which are markedly higher than pCa values observed in frog fibers (i.e., tetanic [Ca^2+^]_i_ is much higher in frog than in mouse fibers) [59]. Thus, changes in myoplasmic P_i_ can affect force generation under the conditions of the present study.

Recently, it was shown that administration of terbutaline (β_2_-adrenoceptor agonist) to humans enhanced muscle performance (both mean and peak power output) during all-out cycling over a 10-s period [39]. Biopsies were taken before and after exercise and analyzed for metabolites associated with energy turnover. From the metabolite values reported in the latter study, we estimated the changes in muscle P_i_ contents during exercise (− 2[ΔATP] − [ΔPCr] − [Δglucose 6-P]). Under placebo conditions, P_i_ increased by 40 mmol/kg dry weight, whereas after inhalation of terbutaline, P_i_ increased by only 7 mmol/kg dry weight. It was suggested that the difference in P_i_ could have contributed to the enhanced performance [39]. Further, it was found that terbutaline also significantly increased anaerobic ATP turnover (10%). These analyses and estimates were based on mixed muscle. The extent to which our findings are applicable to intact humans is unclear. Our results support the idea of lower P_i_ and increased ATP turnover as mechanisms to explain and reflect, respectively, enhanced force generation by activation of β-adrenoceptors during short-term maximal exercise, but it appears that in isolated rodent muscle, decreases in P_i_ account for a small enhancement of muscle performance in EDL in response to β_2_-adrenoceptor activation. Indeed, conversion of the basal P_i_ contents in EDL to mM (divide values in Table 3 by three) yield values of 5.6 mM for control and 4.8 mM for ISO. These concentrations would be expected to decrease force to approximately 70 and 80%, respectively, of that measured in the absence of P_i_ at pH 7.0 [22]. Our observation that the increase in force in the presence of ISO under maximal stimulation conditions was almost 8% (Fig. 2h) agrees well the aforementioned results [22]. This implies that ISO enhances force generation by increasing the amount of force generated per cross-bridge in EDL. In SOL, however, the marked enhancement of force generation is associated with a substantial increase in ATP turnover. This likely reflects an increase in the number of active cross-bridges, which can be attributed to an increase in [Ca^2+^]_i_. Indeed, it appears that under essentially maximal stimulation conditions in SOL, the contractile proteins are not saturated with Ca^2+^ and therefore terbutaline (β-adrenoceptor agonist) administration results in substantial increases in [Ca^2+^]_i_ as well as force [30]. Therefore, our results are consistent with the latter observation, i.e., the increase in [Ca^2+^]_i_ would activate more cross-bridges and thereby increase ATP turnover.

Direct measurements of [Ca^2+^]_i_ were not performed in the present study. However, an indirect way to assess whether there were substantial differences in [Ca^2+^]_i_ between ISO and control treatments in the present study would be to examine the phosphorylase data. Previously, Miller [51] provided evidence to support use of phosphorylase activation in isolated perfused rat hearts exposed to epinephrine (and glucagon) to reflect increases in [Ca^2+^]_i_. Indeed increases in phosphorylase fractional activity in ischemic muscle have been attributed to increases in [Ca^2+^]_i_, which will activate phosphorylase kinase, resulting in phosphorylation of phosphorylase b (see “Results” and [41]). Thus, one can use changes in phosphorylase fractional activity as an indirect marker of changes in [Ca^2+^]_i_. First, it should be noted that activation of β-adrenoceptors does not significantly alter [Ca^2+^]_i_ in isolated glycolytic or oxidative muscle fibers at rest [3, 15, 30]. ISO induced a marked activation of phosphorylase (increase in fractional activity) in EDL at rest, but there was no additional effect in stimulated muscle (Fig. 4). This is likely explained by near maximal activation of phosphorylase during contraction that would preclude detection of additional activation by ISO. An increase in [Ca^2+^]_i_ in EDL under this condition in the presence of ISO is unlikely to have a major effect on force production since troponin C is probably close to saturation with Ca^2+^ already in the absence of ISO [15]. Indeed, our results show that maximum force and force × time integral are enhanced by ISO only by ~ 5%. In contrast, ISO significantly increased phosphorylase fractional activity in SOL during the prolonged contraction, suggesting that there was a significant increase in [Ca^2+^]_i_. The latter is consistent with the observation of increases in [Ca^2+^]_i_ and force in SOL muscle fibers in response to β-adrenoceptor activation under essentially maximal stimulation conditions [30, 58]. Indeed, Ha et al. [30] showed that terbutaline increased force and [Ca^2+^]_i_ by ~ 30% at 50 Hz at 22 °C in isolated SOL fibers. Our findings that maximal force was increased by ~ 25% at 70–100 Hz in SOL muscle at 30 °C (Fig. 2) are in good agreement with the latter findings. Therefore, the current phosphorylase results are consistent with earlier direct measurements of [Ca^2+^]_i_ in SOL fibers, suggesting that [Ca^2+^]_i_ was elevated in SOL fibers in the present study. Nevertheless, direct measurements of [Ca^2+^]_i_ under conditions of the present study are required to confirm this.

Another noteworthy observation is that ISO had a greater effect on muscle glycogenolysis in resting EDL than in resting SOL muscle (Table 3). These findings are consistent with earlier observations that showed a preferential activation of glycogenolysis in resting EDL (glycolytic) vs. SOL (oxidative) muscle in response to administration of adrenaline [17, 37, 57]. In contrast, ISO significantly increased lactate accumulation during continuous stimulation in SOL but not in EDL in the present study. This, too, is consistent with earlier findings showing preferential effects of adrenaline on glycogenolysis in oxidative vs. glycolytic fibers during contraction [27, 57]. In the context of the present study, we propose the following. At rest, energy turnover is low and is not markedly accelerated by β-adrenoceptor activation. Therefore, the minimal degree of glycogenolysis in EDL at rest (relative to contraction) likely occurs for the purpose of trapping P_i_ in hexose phosphates rather than generating ATP. In contrast, the enhancement of glycogenolysis during contraction in SOL primarily occurs to generate more ATP to meet the increased energy demand. Thus, in the context of fight or flight in small mammals, activation of phosphorylase appears to occur for different purposes in oxidative and glycolytic fibers (see below).

Alternative mechanisms to explain the effects of ISO on contractile function under the conditions studied were not investigated in the present study, including activation of Na^+^-K^+^ pumps [20], PKA-dependent phosphorylation of myofilament proteins [49], and trans-sarcolemmal Ca^2+^ influx [25]. These factors, however, were recently reviewed in detail and it was concluded that they were unlikely to explain the β-adrenoceptor-dependent enhanced contractile function of isolated skeletal limb muscles [11] . Similarly, it could be argued that soleus fibers have a higher density of β-adrenoceptors than do EDL fibers [48] and that this accounts for the larger effects of ISO on contractile function in SOL. Although the functional significance of this observation is not fully established, generally the response to β-agonists is greater in fast- than in slow-twitch skeletal muscle [48]. In the present study, we also observed that the ISO-mediated activation of phosphorylase was larger in EDL than in SOL muscles. Further, administration of supraphysiologic doses of adrenaline results in increases in cAMP content of SOL that are slightly less than that seen in EDL, and the activation of phosphorylase is markedly less in SOL than in EDL [17]. These findings do not support the idea that ISO-enhanced contractile function of SOL derives from increased density of β-adrenoceptors.

Based on these observations, we propose a three-phase scenario in the fight or flight response of small mammals. (1) Initially, the perception of a predator will result in enhanced sympathetic discharge in the prey. This will serve as a priming effect in preparation for fight or flight (activation of phosphorylase and decrease in P_i_ primarily in glycolytic fibers). (2) Attack of the predator will result in a supramaximal effort on the part of the prey to avoid being captured. Here, a small enhancement of force (due to decreased levels of P_i_ in glycolytic fibers at the start of the contraction) could prove critical for survival (e.g., a mouse will jump to avoid a pouncing cat). (3) The final phase is escape by a short sprint to safety (e.g., burrow or hole in a wall). This phase would entail enhanced force generation primarily in oxidative fibers that would last several seconds. Indeed, in support of this idea, activation of motor units in SOL fibers at frequencies approaching 100 Hz has been documented in freely moving rats [32]. However, for this scenario to be likely, it would require the existence of a sufficient amount of oxidative fibers that can generate adequate power in the active limbs. With respect to the mouse, it appears that ~ 25–30% of all hind-limb muscles are accounted for by slow oxidative fibers [10]. However, maximal power output in SOL is only about 30% of that observed in EDL at temperatures of 20–25 °C [6, 7, 26, 47]. At more physiological temperatures (30–37 °C), this value approaches 60% [7, 34]. Further, in the fatigued state, maximum power output of EDL decreases by almost 60%, whereas in SOL, it decreases only by ~ 25% [4]. Taken together, these data suggest that the proposed three-phase scenario is feasible.

In conclusion, under conditions of maximal stimulation, ISO has a minor positive effect on force generation in EDL that is associated with a decrease in P_i_ content during onset of contraction. In contrast, ISO has a marked enhancing effect on force generation in SOL that is associated with an increase in glycogenolysis and ATP turnover. Thus ISO-mediated activation of phosphorylase contributes to enhanced performance of SOL and EDL muscles via separate mechanisms.
